# Extraosseous Ewing's Sarcoma of the Pancreas: An Uncommon but Treatable Disease

**DOI:** 10.7759/cureus.1882

**Published:** 2017-11-26

**Authors:** Muhammad W Saif, Kristin Kaley

**Affiliations:** 1 Hematology/Oncology, Tufts Medical Center; 2 Cancer Center, Lexington Medical Center

**Keywords:** sarcomatoid carcinoma, neuroectodermal tumor, cytogenetics, pancreas, ewing's sarcoma

## Abstract

Extraosseous Ewing's sarcoma/primitive neuroectodermal tumor (ES/PNET) is a rare but aggressive and malignant tumor, and has been reported in various sites such as the lungs, biliary tract, kidney, prostate, stomach, esophagus, oral cavity, salivary glands, urinary bladder, uterus, cervix, gonads, and vagina. However, the pancreas is considered to be an extremely uncommon site and only a handful of cases have been published to date. We present here another case of a pancreatic ES/PNET. Our case intensifies the importance to recognize this rare type of tumor in the pancreas as there is a broad spectrum of tumors with a similar morphology that includes sheets of small, round blue cells. As observed in our present case, this problem is markedly challenged when the tumor site of origin is uncertain.

## Introduction

Ewing's sarcoma (ES) was first described by James Ewing in 1921 and the extraosseous Ewing's sarcoma/primitive neuroectodermal tumor (ES/PNET) was first described by Tefft in 1969 [1-2]. ES and PNET share the same cytogenetic alterations (t(11;22) (q24;Q12) which forms the EWSR1-FLI1 fusion product) as well as similar histopathological and immunohistochemical characteristics. Therefore, they are classified under the same group of lesions as the ES/PNET family of tumors. ES/PNET consists of the following four sub-types: Ewing's sarcoma of bone (ESB), extraosseous Ewing's sarcoma (EES), peripheral primitive neuroectodermal tumor (pPNET), and Askin's tumor.

Since its specific characterization in the 1980s as sharing the same cytogenetic alterations, ES/PNET has been published in many case reports and series indicating sites of unusual occurrence such as the oral cavity, salivary glands, lung, heart, pericardium, esophagus, stomach biliary tract, pancreas, prostate, kidney, urinary bladder, uterine corpus, cervix, gonads, and vagina [3]. Up to a dozen case reports of EES/pPNET primary located in the pancreas were found in the review of the literature and here we add another case of a 38-year-old woman to the list [4-9].

## Case presentation

**Figure 1:**
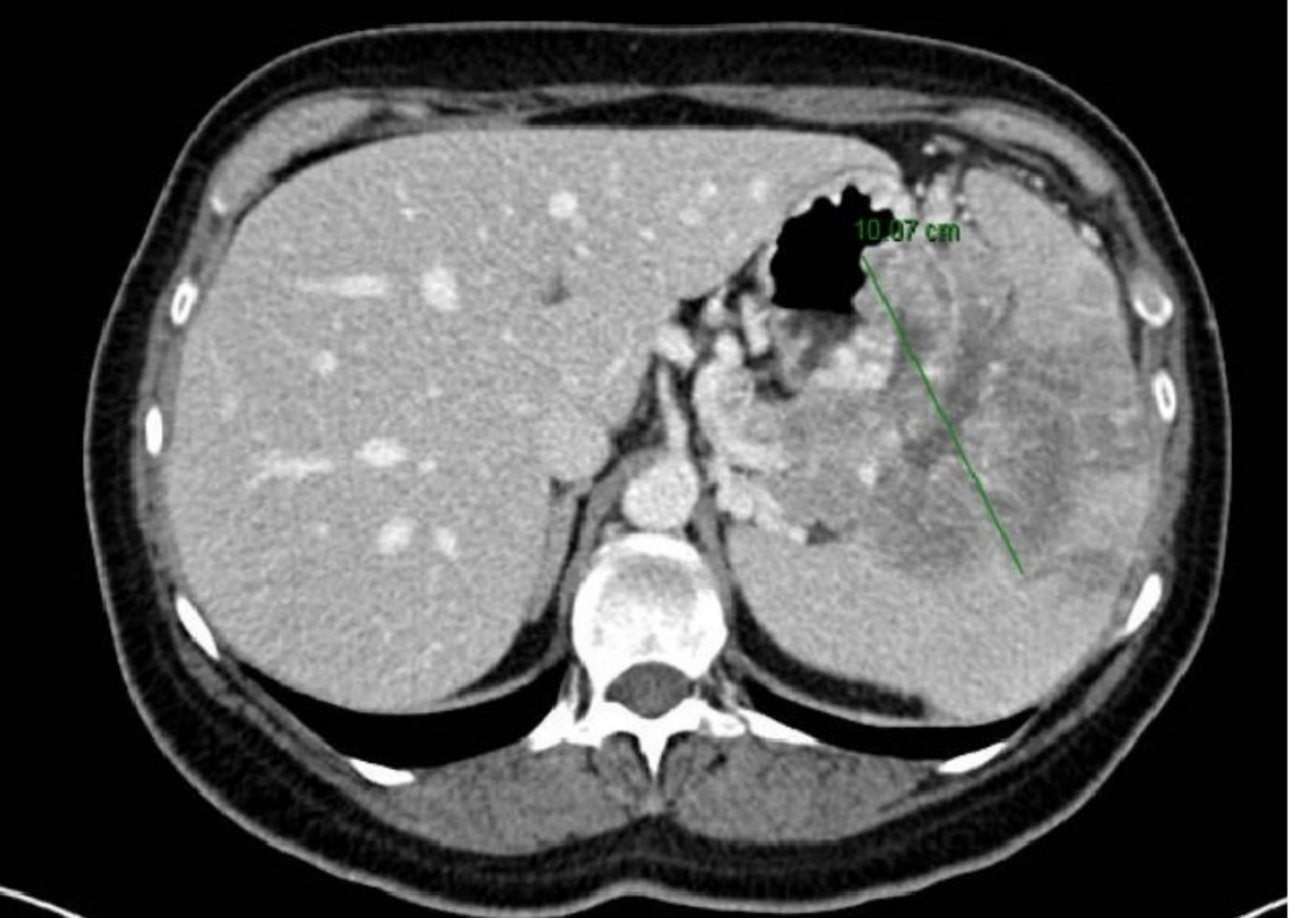
Computed tomography (CT) scan of the abdomen showing a big mass in the body and tail of the pancreas CT scan examination showed an 8 x 10 cm mass in the body and part of the tail of the pancreas with hypodense non-enhancing areas suggestive of necrosis or cystic change.

A 38-year-old woman presented with abdominal pain of 2-3 weeks duration. There was no associated vomiting, diarrhea, or weight loss. A physical examination revealed only mild epigastric tenderness. No organomegaly was noted. Hemoglobin was 12.1 g/dL. All other laboratory parameters were within normal limits. Computed tomography (CT) examination showed an 8 x 10 cm mass in the body and part of the tail of the pancreas with hypodense non-enhancing areas suggestive of necrosis or cystic change [Figure 1].

The patient underwent distal pancreatectomy along with splenectomy. Grossly, the tumor was well-circumscribed, partly encapsulated, and the cut section revealed hemorrhages and areas of necrosis. Microscopic examination showed sheets of small round cells with enlarged round to oval nuclei, fine stippled chromatin, and moderately clear to amphophilic cytoplasm, which was periodic acid-Schiff stain positive. Geographic areas of necrosis with focal peritheliomatous proliferation of tumor cells around the blood vessels, increased mitosis, prominent apoptosis, and nuclear moulding were also noted. The tumor cells were CD99 positive, while cytokeratin (CK), desmin, synaptophysin (SYP), and chromogranin (CHR) were negative. Based on the morphology and immunohistochemistry findings, a final diagnosis of ES/PNET of the pancreas was made. Cytogenetics showed alterations at t(11;22) (q24;Q12).

The metastatic workup including a CT scan of the abdomen, pelvis, and chest of the patient was negative. She received two cycles of alternating IE (ifosfamide and etoposide) and VAC (vincristine, adriamycin, and cyclophosphamide) chemotherapy. Currently, the patient is receiving regular follow-up care and has no evidence of cancer at six months post-adjuvant chemotherapy.

## Discussion

Our case of EES/pPNET arising in the pancreas underlines the importance of including this entity in the differential diagnosis. Gross pathology showed that ES/PNET is a poorly differentiated tumor which morphologically appears as "small round cells", mimicking with other tumor types such as lymphoma, pancreatic neuroendocrine tumor (pNET), pancreatoblastoma, extra-renal Wilm's tumor, extra-adrenal neuroblastoma, hepatoblastoma, rhabdomyosarcoma, desmoplastic small round cell tumor (DSRCT), and visceral small cell neuroendocrine carcinoma (SCNC) [1-9]. It is the characteristic translocations that disrupt the EWSR1 gene located at 22q12 which confirms the diagnosis and is found in 95%-100% of these cases [3].

Unlike the Ewing intraosseous tumor which is more commonly seen in males, there is no gender predilection in ES/PNET. More than 70% of the patients are diagnosed during their teenage years [3-7]. Almost all patients have occult disseminated disease at the time of diagnosis [3-9]. There are no specific signs and symptoms except the location of the primary site. Though not specific, the delineation of this type of tumors is usually in the paravertebral region, retroperitoneal area, thoracic wall, and lower extremities. There are no characteristic radiological signals; however, most lesions are encapsulated, hypoechoic on ultrasound, hypodense on CT, and iso-hypointense on T1, hyperintense on T2 on magnetic resonance imaging (MRI). Finally, the diagnosis is confirmed with cytogenetics and immunohistochemistry.

Improvements in the multimodality treatment of ES/PNET have shown a dramatic improvement in the clinical outcome. As mentioned previously, the majority of the cases have occult or evident metastasis, hence multidrug chemotherapy as well as local disease control with surgery and/or radiation that is indicated in the treatment of all patients. It is crucial that a patient diagnosed with these tumors is treated at a facility which has an interdisciplinary team of experts who have massive experience handling these tumor types. This list may include but not be limited to medical oncology, radiation oncology, orthopedics, musculoskeletal radiology, musculoskeletal pathology, spine surgeons, vascular surgeons, and plastic surgeons.

Prior to the inclusion of multi-agent chemotherapy, the long-term survival of ES was dismal (less than 10%). However, with the advent of intensive chemotherapy regimens and multimodality care, these numbers have improved to 60% and 70% for patients with long-term survival. This fact also proves the fact that ES is a chemo-sensitive malignancy. The most commonly used chemotherapeutic agents include doxorubicin, cyclophosphamide, vincristine, actinomycin-D, ifosfamide, and etoposide, which have been refined into regimens. Grier HE, et al. led the Intergroup Ewing Sarcoma Study III and randomized patients to treatments with a three-drug therapy (VDC: vincristine, doxorubicin, and cyclophosphamide) versus a five-drug therapy (IE: ifosfamide and etoposide in addition to VCD) [10]. VDC was administered over two days followed by ifosfamide and etoposide given over five days. The two combinations (VDC and IE) are traditionally alternated every three weeks. As reported in the New England Journal of Medicine, patients receiving a five-drug regimen versus a three-drug regimen had an improved survival of 72% vs. 61% (p = 0.01). This study defined the five-drug regimen as the gold standard chemotherapy regimen for this family of tumors. Dactinomycin is no longer used in the United States but continues to be used in the Euro-Ewing studies. Increased dose intensity of doxorubicin during the initial months of therapy was associated with an improved outcome in a meta-analysis done prior to the standard use of ifosfamide and etoposide. The 10-year event-free survival has been reported to be around 51% with VAC plus ifosfamide.

Radiation therapy has a role in these patients, particularly in those patients with residual disease after surgery and chemotherapy. Hyperfractionated radiation therapy has not shown to be of any extra benefit; however, proton-beam radiation therapy and intensity-modulated radiation therapy (IMRT) treatment seem to be promising but lack randomized data.

## Conclusions

Extraosseous ES/PNET is a rare entity but poses a diagnostic dilemma to physicians as well as pathologists. Moreover, early recognition of ES/PNET can produce the best chance of survival with current treatment modalities. This problem is significantly critical when the tumor site of origin is uncertain or rare, such as the pancreas, as in the case of our patient. Therefore, extraosseous ES/PNET should be considered in the differential diagnosis of intraabdominal and extraintestinal masses, especially in patients with no specific signs and symptoms. The timely use of histomorphology and immunohistochemistry can aid in securing the diagnosis and lead to an earlier treatment for a better outcome.
